# *In Vivo* Differentiation of Mesenchymal Stem Cells into Insulin Producing Cells on Electrospun Poly-L-Lactide Acid Scaffolds Coated with *Matricaria chamomilla* L. Oil

**DOI:** 10.22074/cellj.2016.4558

**Published:** 2016-08-24

**Authors:** Afsaneh Fazili, Soghra Gholami, Bagher Minaie Zangi, Ehsan Seyedjafari, Mahdi Gholami

**Affiliations:** 1 Department of Anatomy, School of Veterinary Medicine, University of Shiraz , Shiraz, Iran; 2 Department of Histology, Medical Sciences Faculty, Tehran University of Medical Sciences, Tehran, Iran; 3 Department of Biotechnology, College of Science, University of Tehran, Tehran, Iran; 4 Faculty of Pharmacy and Pharmaceutical Sciences, Research Center, Tehran University of Medical Sciences, Tehran, Iran

**Keywords:** Differentiation, Transplantation, Scaffold, Insulin

## Abstract

**Objective:**

This study examined the *in vivo* differentiation of mesenchymal stem cells (MSCs) into insulin producing cells (IPCs) on electrospun poly-L-lactide acid (PLLA) scaffolds coated with Matricaria chammomila L. (chamomile) oil.

**Materials and Methods:**

In this interventional, experimental study adipose MSCs (AMSCs) were isolated from 12 adult male New Zealand white rabbits and characterized by flow cytometry. AMSCs were subsequently differentiated into osteogenic and adipogenic lines. Cells were seeded onto either a PLLA scaffold (control) or PLLA scaffold coated with chamomile oil (experimental). A total of 24 scaffolds were inserted into the pancreatic area of each rabbit and placement was confirmed by ultrasound. After 21 days, immunohistochemistry analysis of insulin-producing like cells on protein levels confirmed insulin expression of insulin producing cells (IPSCs). Real-time polymerase chain reaction (PCR) determined the expressions of genes related to pancreatic endocrine development and function.

**Results:**

Fourier transform infrared spectroscopy (FTIR) results confirmed the existence of oil on the surface of the PLLA scaffold. The results showed a new peak at 2854 cm^-1^ for the aliphatic CH_2_ bond. *Pdx1* expression was 0.051 ± 0.007 in the experimental group and 0.009 ± 0.002 in the control group. There was significantly increased insulin expression in the scaffold coated with chamomile oil (0.09 ± 0.001) compared to control group (0.063 ± 0.009, P≤0.05). Both groups expressed *Ngn3* and *Pdx1* specific markers and pancreatic tissue was observed at 21 days post transplantation.

**Conclusion:**

The pancreatic region is an optimal site for differentiation of AMSCs to IPCs. Chamomile oil (as an antioxidant agent) can affect cell adhesion to the scaffold and increase cell differentiation. In addition, the oil may lead to increased blood glucose uptake in pathways in the muscles, liver and fatty tissue of a diabetic animal model by some probable molecular mechanisms.

## Introduction

Stem cell therapy can provide an alternative approach for repair and regeneration of tissues and organs (1). These cells are able to replicate, transform and differentiate into different cell types (2). Mesenchymal stem cells (MSCs) are promising candidates for cell-based therapies (3). Bone marrow and adipose tissue are two suitable sources of MSCs (4). Adipose MSCs (AMSCs) are conveniently accessible and have the ability to differentiate into multiple cell lines (5). 

Due to their capacity to generate multiple cell lines, MSCs can be affected by certain conditions *in vivo* or *in vitro* which can influence their conversion to cells with specific functions such as heart muscle cells or insulin producing cells (IPCs) in the pancreas (6). 

Regenerative medicine is highly dependent on three-dimensional cultivation techniques on scaffolds (7), which are supporting structures similar to the cell matrix (8). Poly (L-lactide) acid (PLLA) and gelatinous scaffolds are considered to be two important biocompatible, dissoluble polymers used in tissue engineering (9). The three-dimensional microenvironments supplied by these scaffolds provide stable structures for transplanted cells (10), creating an environment similar to secretion of the extracellular matrix (11). The MSCs-PLLA scaffold system can be used as cell therapy for treatment of chronic diseases such as diabetes (12). 

Traditional medicine, which includes the use of different herbs, is another option for treatment of certain diseases. Herbal remedies have gained considerable interest and researches on the potential of these agents as treatment and control of numerous diseases is an expanding, promising area. Traditionally, topical agents have been used to control diabetic lesions (13). However, the formulae of these herbal medicines are too diversified (14). More importantly, the lack of good systematic documentation of clinical data relevant to evidence-based scientific support has decreased their acceptability worldwide. The herb *Matricaria chamomilla* L. (chamomile), contains a number of properties for pain relief, anti-anxiety treatment, and treatment of oral and dermal wounds (15). Chamomila decrease blood sugar levels, increasing glycogen stores in the liver and red blood cells contained sorbitol (16). 

The main goal of the present study is to investigate the properties of chamomile oil and the tissue engineering effect on differentiation of AMSCs to IPCs by mimicking the environment of the pancreatic region in healthy rabbits. This study may assist with the development of a new approach to treat diabetic patients based on cell therapy and traditional medicine. 

## Materials and Methods

### Animals

A total of 30 male New Zealand white rabbits (2.0-2.5 kg, Razi Institute, Iran), 10 weeks of age, were used in this experimental study. All experimental procedures were approved by the Animal Ethics Committee of Shiraz University and performed in accordance with the National Institute of Health Animal Care Guidelines. All rabbits received food and water ad libitum and were kept in a room controlled for temperature (22 ± 2˚C) and humidity. Rabbits were mainw tained a 12:12 hour light and dark cycle. 

### Isolation and culture of adipose-derived cells

Adipose tissues were isolated from rabbits previously anesthetized with 40 mg/kg ketamine and 5 mg/kg xylazine intraperitoneal (IP). In each animal, a midline abdominal incision was made after which we harvested approximately 100 ml of adipose tissue from the perivesical region. The adipose tissue was minced in cold phosphate-buffered saline (PBS, pH=7.4), then digested with 0.1% collagenase type I (Sigma, Germany) for 90 minutes at 37˚C with modf erate shaking. The cell suspension was centrifuged at 1000 rpm for 4 minutes. We discarded the supernatant and suspended the pellet in 20 ml of the culture medium that consisted of 4.5 g/l glucose-Dulbecco’s modified Eagle’s medium (DMEM, Gibco, Germany) supplemented with 15% regular fetal bovine serum (FBS, Gibco, Germany), and 1% penicillin-streptomycin (Gibco, Germany). The suspended cells were filtered through 75 μm nylon mesh filters (BD Biosciences, USA). Finally, 5 ml of the cell suspension was seeded into 60-mm culture dishes (Grainer, Germany). The cells were incubated at 37˚C in a humid environment with 5% CO_2_for 7 days. The medium was changed every day to remove any nonattached cells. 

### Flow cytometry analysis of adipose mesenchymal stem cells

The AMSC cell surface antigen profile was characterized by flow cytometry as described previously (17). Briefly, AMSCs were trypsinized and washed with cold PBS that contained 1% fetal calf serum (FCS) at approximately 1×10^6^cells in 50 μl of PBS. samples were placed on ice and separately labeled with optimal dilutions of fluorescein isothiocyanate-conjugated monoclonal antibodies (Abcam, Germany) against CD44 and CD45 (Abcam, Germany) Then incubated in the dark for 30 minutes, cells were washed with cold PBS that contained 1% bovien serum albumin (BSA). Nonspecific fluorescence was determined by incubating cells with the isotype-matched antibody. At least 10000 events were collected from each run of flow cytometry. Data were analyzed using Cell Quest software (Becton Dickinson, Germany). 

### Adipogenic and osteogenic differentiation of adipose mesenchymal stem cells

Adipogenic and osteogenic differentiation of AMSCs were induced according to a previously reported procedure (18). Briefly, for adipocytic differentiation we grew AMSCs for 2-4 weeks in 1 mM dexamethasone, 10 μg/ml insulin, and 0.5 mM isobutylxanthine (Sigma, Germany) in alpha MEM medium that contained 10% FBS. Oil red O staining was used for adipocytes according to standard techniques. 

AMSCs were induced to osteogenic differentiation over a 3-4 week period in osteogenic medium that consisted of AMSCs growth medium supplemented with 100 nM dexamethasone, 0.05 mM ascorbic acid, 10 mM β-glycerophosphate, and 10 nM 1α,25-dihydroxyvitamin (Sigma, Germany). Alizarin red S (ARS, Millipore, Germany) staining was used for osteoblasts according to standard techniques. 

### Scaffold fabrication

PLLA was dissolved in chloroform (Merck, Germany) and combined with dimethyl formamide (DMF, Sigma Aldrich, Germany) at a ratio 4.25:0.75. The polymer so-lution was loaded into 5 ml plastic syringes and connected to a 21-gauge needle. A positive voltage between the needle and collector was applied, after which one of the mats was immersed overmight in oil, rinsed, and used for further experiments. 

### Hydrophilicity testing

We measured the water contact angle of the scaffolds’ surfaces at room temperature by the sessile drop method with a G10 contact angle goniometer (Kruss, Germany). 

### Fourier transform infrared spectroscopy

Coating of the oil on the surface of the scaffolds was investigated by Fourier transform infrared spectroscopy (FTIR). Infrared spectra were assessed by a Vertex 80 spectrometer with a DTGS detector. 

### Cell seeding on the scaffold

Scaffolds were inserted in DMEM supplemented with 10% FBS and incubated overnight at 4˚C. AMSCs were isolated from rabbit adipose tissue. Passage three AMSCs were seeded onto scaffolds (with or without oil coating) at a density of 1×10^5^cells per well of 24-well plates in DMEM supplemented with 10% FBS, penicillin and 1% streptomycin and incubated at 37˚C and 5% CO_2_. Cell seeding was checked after one day by propidium iodide (PI) staining. We added a pure concentration of oil to the scaffold 24 hours before seeding the cells at 37˚C. 

### Scanning electron microscopy

We investigated the surface morphology of the fabricated scaffolds by cutting them into 1×1 cm^2^samples which were gold sputtered in a vacuum. Images were visualized by an EM3200 digital scanning electron microscope (KYKY, China). 

### Incorporation of adipose mesenchymal stem cells into the poly (L-lactide) scaffold

The PLLA was sterilized with ethylene oxide prior to cell seeding. The PLLA was placed into a 24-well plate and soaked with cell culture liquid. Passage three AMSCs at an adjusted cell density of 1×10^6^/ml were divided into two groups, control and experimental. In the cell+scaffold group (experimental) a 200 μl cell suspension was poured onto each scaffold. Next, an additional 2 ml cell suspension fluid (cell density: 1×10^6^/ml) was added to each well. In another group, the scaffold was used alone in the body. The experimental and control groups were placed into the cell culture box (37˚C, 5% CO_2_, and 95% humidity). The cell culture solution was replaced every 3 days. 

### Differentiation of adipose mesenchymal stem cells toward pancreatic progenitor cells by mimicking

the pancreatic microenvironment *in vivo* The AMSC with the scaffold was inserted in the caudal of the stomach located between the pancreas and spleen for 21 days. In each group, 12 coated and 12 uncoated scaffolds were implanted into healthy rabbits. AMSCs were autografted in order to prevent an immune reaction. Ultrasound was used for implantation of the scaffold. After 21 days, the scaffold was removed and prepared for differentiation assessment by immunohistochemistry and quantitative polymerase chain reaction (qRT-PCR). 

### Quantitative analysis of gene expressions

We performed qRT-PCR to assess specific gene expressions after *in vivo* cell differentiation. The animals were sacrificed and both experimental and control group scaffolds were removed, and homogenized in microtubes that contained RNX plus (Cinnagen, Iran). Total RNA was extracted from differentiated pancreatic-like cells using RNAX plus according to the manufacturer’s recommendations. In order to eliminate genomic contamination, RNA was treated with DNase I using a kit (EN0521, Fermentas, Germany). RNA concentrations were measured by spectrophotometry (Eppendorf, Germany). The cDNAs were made from 1000 ng DNase-treated RNA samples using a RevertAid™ First Strand cDNA Synthesis kit (Fermentas, Germany) according to the manufacturer’s protocol. Primers were designed by the NCBI website and synthesized by Pishgam Company (Table 1). PCRs were performed using Master Mix and SYBR Green I in an Applied Biosystems, StepOne™ thermal cycler (Applied Biosystems, USA). The PCR program began with an initial melting cycle for 5 minutes at 95˚C to activate the polymerase, followed by 40 cycles of melting (30 seconds at 95˚C), annealing (30 seconds at 58˚C), and extension (30 seconds at 72˚C). The quality of the PCR reactions was confirmed by melting curve analyses. For each sample, we amplified both the reference (*Gapdh*) and target genes in the same run. Reference genes were approximately equal.

**Table 1:** Primers used for qRT-PCR


Genes	Primer sequences (5´-3´)	Length

*Pdx1*	F: 5´GAAGATGCTGGTGGACCTTCTG3´	111 bp
	R: 5´AGGTGCATCACAATGGCAGAC3´	
*Insulin*	F: 5´TCGTCAACCAGCACCTGTGC3´	115 bp
	R: 5´ACCTGCAGCTCCTCCACCTC3´	
*Gapdh*	F: 5´CACCCACTCCTCTACCTTCG3´	116 bp
	R: 5´GGTCTGGGATGGAAACTGTG3´	


qRT-PCR; Quantitative polymerase chain reaction.

### Immunohistochemistry

The animals were sacrificed 21 days after transplantation. The scaffolds were removed, sectioned, and fixed in 4% neutral buffered paraformaldehyde. Antibodies specific for *Pdx1* (1:100, AB3209, Chemicon, UK), *Ngn3* (1:100, ab13840, Abcam, UK), secondary anti-rabbit IgG-FITC, and anti-rabbit IgG-PE (Abcam, UK) were used. Images were captured using a Zeiss LSM 5 fluorescent microscope. 

### Statistical analysis

We used the Statistical Package for the Social Sciences (SPSS 16.0) for data analysis. Statistical significance was assessed by oneor two-way analyses of variance and Tukey’s multiple comparison tests. A confidence level of 95% (P<0.05) was considered statistically significant. All data have been presented as mean ± SD. 

## Results

### Culture characteristics

**Fig.1:**
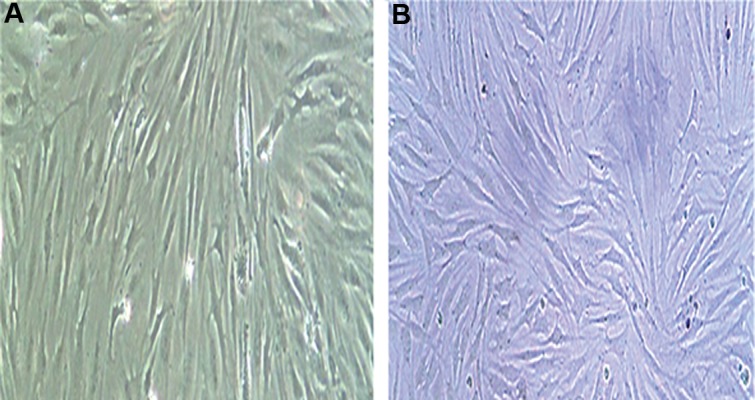
Morphology of adipose mesenchymal stem cells (AMSCs) varies according to the culture time. A and B. Morphology of AMSCs after the third passage.

There were attached spindle shaped cells observed two days after primary cultivation (Fig.1A). Expanded cells grew during 10 days into a homogenous population. These fibroblast-like cells formed Confluent colonies in the flask by day 15 (Fig.1B). The rate of cell growth changed minimally after an increase in the number of passages with no morphological changes observed. 

### Differentiation assay

**Fig.2:**
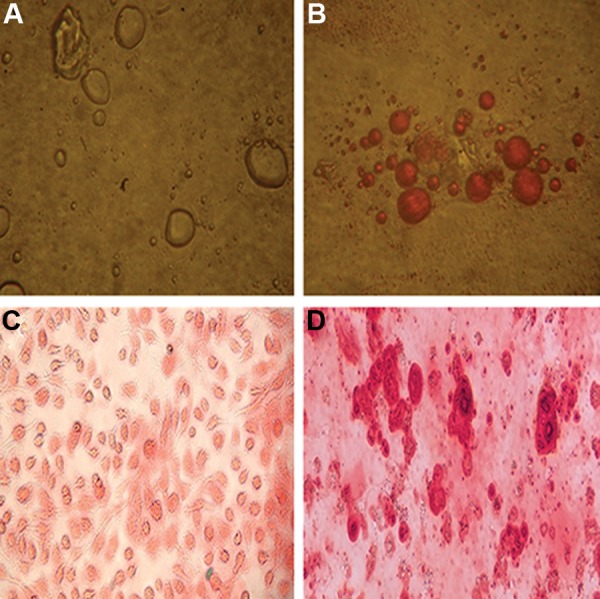
Osteogenic and adipogenic differentiation. A. Adipocytes differentiated from mesenchymal stem cells (MSCs), B. Lipid vacuoles stained with oil red O after differentiation to adipocytics and, C and D. Alizarin red staining (ARS) of calcium deposits after osteoblastic differentiation.

Adipogenic differentiation was confirmed by morphological observation and oil red O staining. Two weeks from the beginning of differentiation, the cells showed a few vacuoles that enlarged in size and number by increased passages up to 3 weeks (Fig.2A,B). There were minimal depositions in the cytoplasm of the AMSC derived osteoblasts 3 weeks after seeding (Fig.2C). Mineralization was confirmed by ARS (Fig.2D). These results showed the multi-potential capability of AMSCs to form other mesenchymal lineages. 

### Flow cytometric analyses

Flow cytometric analyses of the isolated cells showed that they were positive for CD44 (93.52%) and negative for hematopoietic antigens such as CD45 (3.87%). This result indicated the purity of the MSCs during cell cultivation after the third passage (data not shown). 

### Morphological structures of the poly (L-lactide) scaffold

**Fig.3:**
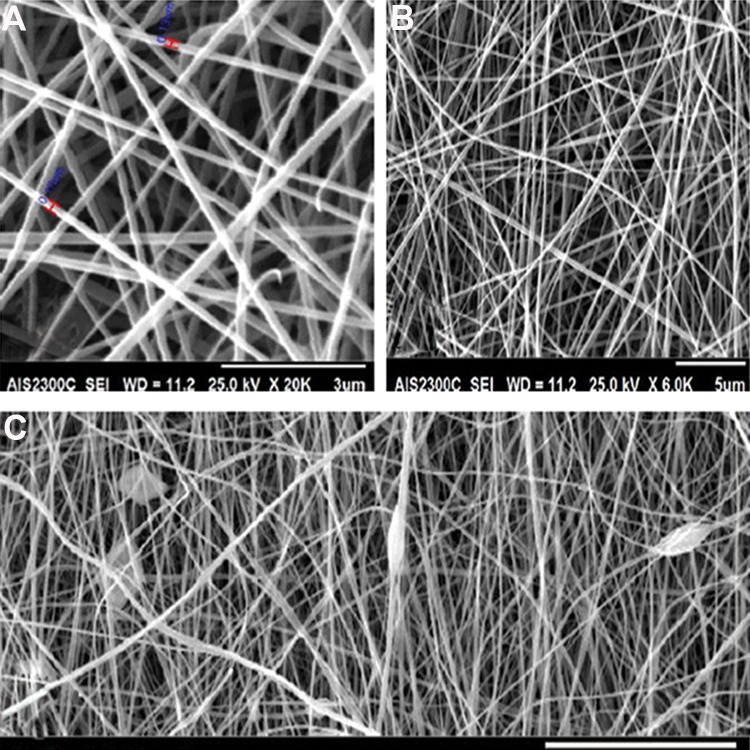
Scanning electron micrograph (SEM) images show the electrospun poly (L-lactide) acid (PLLA) scaffold. A. 30 µm, B. 5 µm, and C. 3 µm. The picture rep represents fiber diameters of the PLLA scaffold.

**Fig.4:**
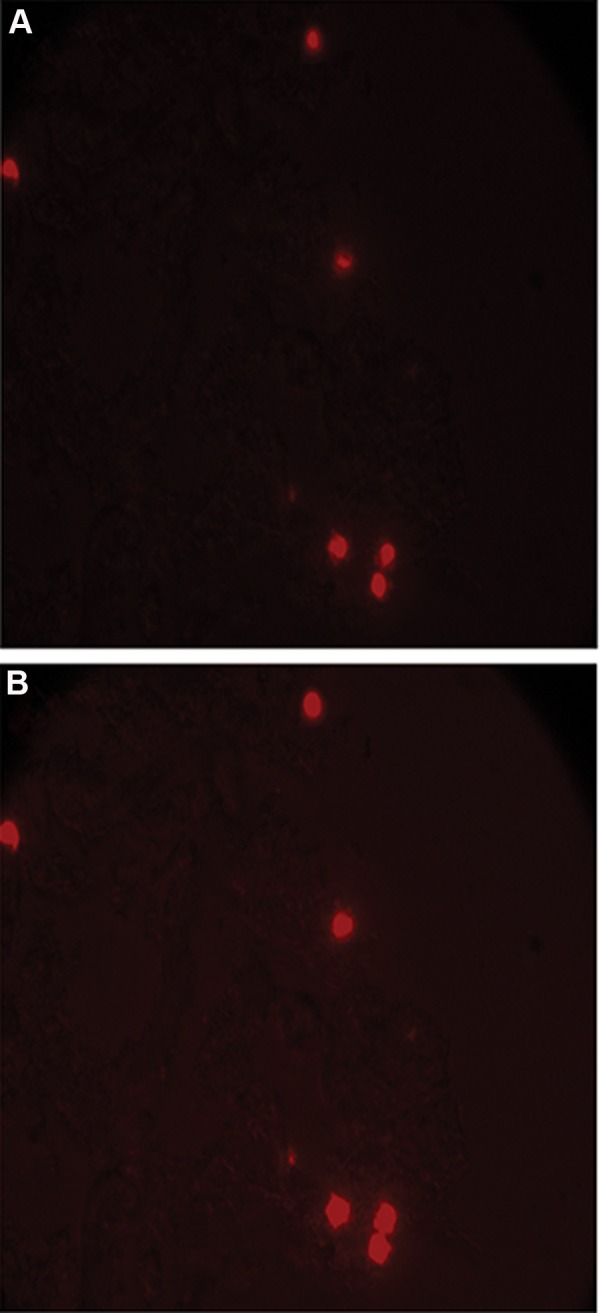
Presence of cells on the poly (L-lactide) acid (PLLA) scaffold. A. The cells were stained by PI and B. Merged cells and scaffold (magnification: ×200). PI; Propidium iodide.

Morphological structures of the poly (L-lactide) scaffold Scanning electron microscopic (SEM) images showed the electro spun PLLA (Fig.3). SEM images demonstrated that when AMSCs were seeded on scaffolds with a parallel pattern, the fibers conducted the cell’s elongation through themselves. There was no observable difference between un-coated and chamomile oil coated scaffolds in terms of cell adherence and growth after one day of cell seeding. PI staining showed presence of cells on the coated and uncoated scaffolds after one day of cell seeding (Fig.4). 

### Fourier transform infrared spectroscopy assessment

**Fig.5:**
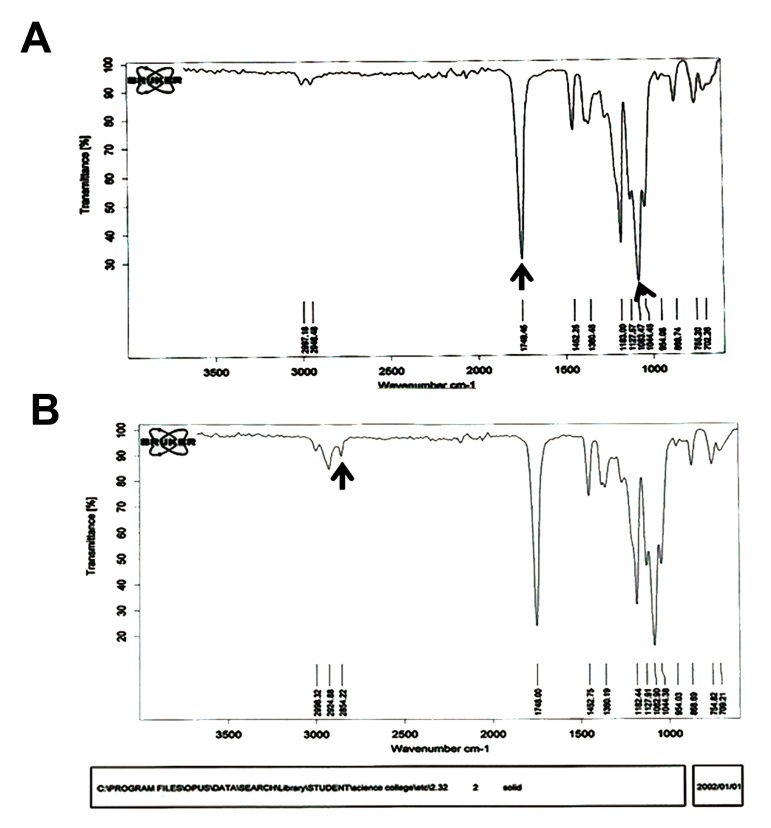
Fourier transform infrared spectroscopy (FTIR) of poly (L-lactide) acid (PLLA) scaffold. A. FTIR of PLLA scaffold and B. PLLA coated by oil.

FTIR was performed to confirm the existence of the chamomile oil on the surface of the PLLA blends (Fig.5). The peak at 1082 cm^-1^corresponded to a C-H stretch, whereas at 1748 cm^-1^, the peak corresponded to a C=O bond (Fig.5A). The results showed the presence of a new peak at 2854 cm^-1^which confirmed the features of oil for an aliphatic CH^2^bond (Fig.5B). 

### In vivo transplantation of poly (L-lactide) scaffold+chamomile oil loaded by adipose

**Fig.6:**
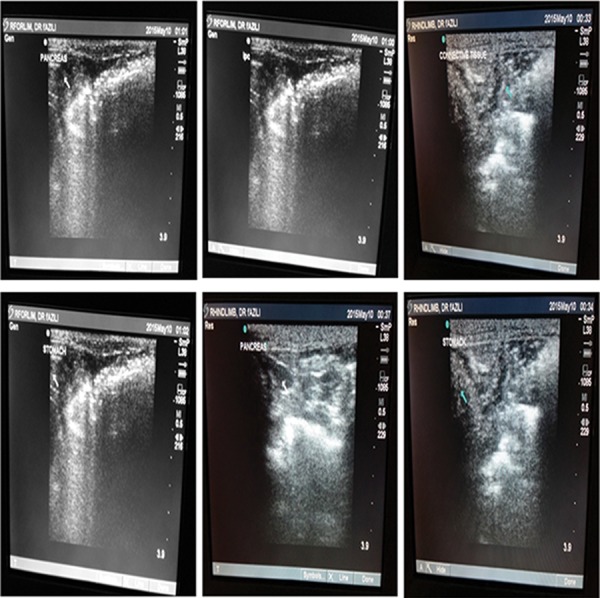
Ultrasound images for assessment of scaffold location after implantation in caudal of stomach. White arrow shows site of implantation near to the pancreas as a heteroechogenic mass.

Ultrasound imaging was used to assess the implantation scaffold. Based on our observation, the scaffold coated with chamomile oil loaded by AMSCs was visualized as a heteroechogenic mass located near the pancreatic region in the caudal of the stomach, between the spleen and pancreas. This finding confirmed the proper location of the scaffold following transplantation (Fig.6). The PLLA scaffold can remain up to 24 months inside the body. We have used this scaffold for 21 days in the rabbit as a carrier of AMSCs during *in vivo* differentiation. 

### Quantitative polymerase chain reaction analysis of gene expression of insulin producing cells

**Fig.7:**
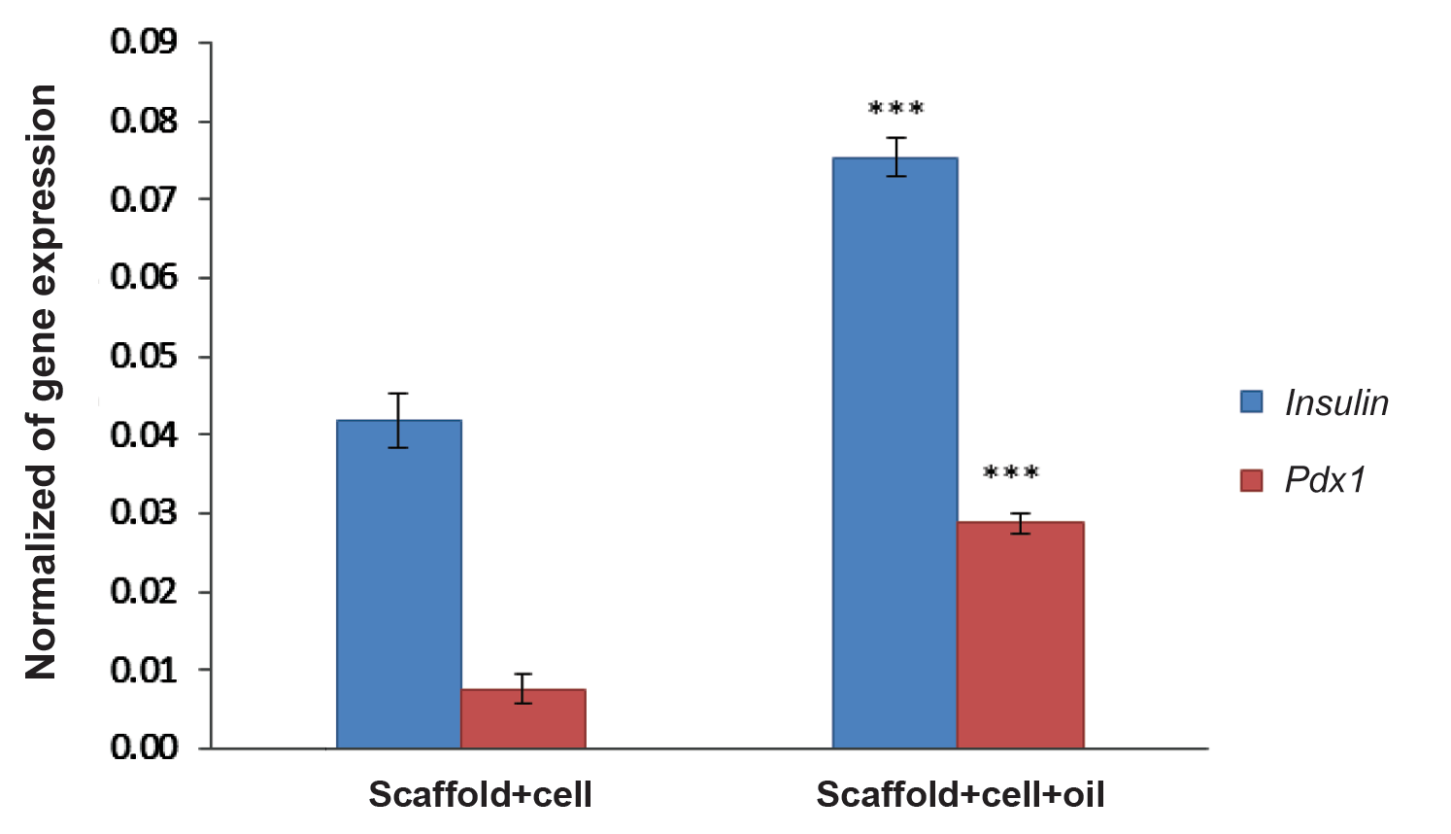
Real time-PCR analysis. Profile of mean normalized specific insulin producing cells (IPCs) (y-axis) shown in the different groups (xaxis) for derivation of IPCs. mRNA levels were normalized with respect to *Gapdh*, chosen as the internal controls. Histograms show mean expression values ± SD (n=3, P<0.05). ***; Significant difference with other group in the same genes and PCR; Polymerase chain reaction.

We sought to determine whether the AMSCs differentiated into pancreatic endocrine cells. QRT-PCR was performed to confirm expressions of the genes related to pancreatic endocrine development and function. Results from a representative experiment are shown in Figure 7. *Pdx1* was expressed 0.009 ± 0.0002 in the scaffold+cell group and 0.051 ± 0.0007 in the scaffold+cell+oil group. Also, Insulin was expressed 0.063 ± 0.009 in the scaffold+cell group compared to 0.09 ± 0.001 in the scaffold+cell+oil. These results showed a significant difference between the two groups in expressions of these specific genes (P≤0.05). 

### Immunocytochemistry for insulin

**Fig.8:**
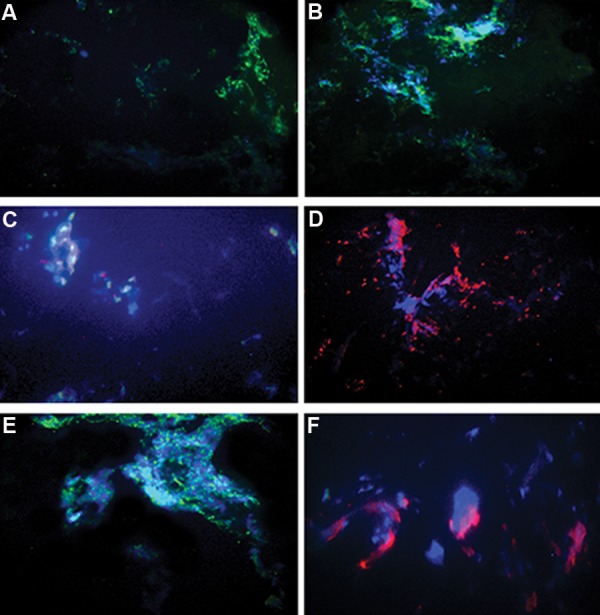
Immunostaining for *Pdx1* and *Ngn3* proteins in adipose mesenchymal stem cells (AMSCs) derived insulin producing cells (IPCs) after 21 days of *in vivo* transplantation. A and B. Ngn3-FITC, C and D. Pdx1-PE. Nuclei were stained with DAPI, E and F. Normal pancreas.

To confirm the insulin expression of the IPCs at the protein level, we conducted immunocytochemistry analyses of the differentiated AMSCs. Figure 8 shows that the differentiated cells with islet-like structures were positive for *Pdx1* and *Ngn3*. Pancreas tissue was used as the positive control. 

## Discussion

Our findings suggested that AMSCs could successfully differentiate into IPCs, which resulted in cells that were morphologically similar to pancreatic islet cells (19, 20). These cells had the capability to express insulin as confirmed by Dithizone (DTZ) staining and immunohistochemistry (21). qRT-PCR and immunohistochemistry analyses showed that the differentiated cells expressed *Pdx1, Ngn3* and *Insulin*.

The present study showed the potential *in vivo* effect of a poly (L-lactic)-collagen-based scaffold coated by chamomile oil on the differentiation of rabbit AMSCs into IPCs. This approach has been used for the first time. The results indicated that *in vivo* transplantation within the pancreatic region could be effective on differentiation of AMSCs to IPCs without the need for extra supplements such as certain growth factors. Chamomile oil, as a traditional treatment for diabetics, was useful in the improvement of this process (16, 22).

Another branch of stem cell therapy involves the *in vitro* generation of graftable tissues that combine cells (normal, manipulated or engineered) or their components with scaffolds to generate three-dimensional implants (23). To reach this goal, methods that combine scaffolds with growth factors (24), transfection of gene vectors in matrices (25), and the combination of cells and scaffolds have been considered (26). Scaffolds were used in treatment of certain types of injures (24).

Electrospun scaffolds consist of a network of structures with a large surface region that mimics structures of the normal extracellular matrix (ECM) microenvironment (10). Surface coating uses various biomaterials to change the mechanical foundation, bioactivity, cytocompatiblity, and hydrophilicity of the scaffolds (27-29). PLLA has been well investigated due to its physical, chemical, morphological, and thermal properties. A number of researches on the surface manipulation of PLLA through the gelatin coating process showed that the hydrophilicity, physical property, and cellular adhesion and differentiations of the scaffold had greater augmentation after these treatments (30, 31). PLLA coated by chamomile oil and its blends did not have any side effects on the bioactivity or antigenicity of the scaffold Ease of use encouraged us to modify the surface of blends by coating with chamomile oil. Of note, the oil perfectly covered the nanofibers of the scaffold without any morphological changes in fiber thickness, shape and size of the cavities, or scaffold alignments.

AMSCs have been used as a cell source to seed on PLLA coated by oil. The morphological properties of AMSCs on PLLA were observed based on nuclei staining. SEM observation also showed pores and lines of PLLA fibers. In a comparison of the oil coated versus non-coated hybrids, we observed in the SEM images that surface oil modification did not bend the topographic guidance of the fibers which indicated that oil coating maintained scaffold bioactivity. The nuclei PI stained to confirm the location of the cells on the oil coated scaffold. The measurements of the contact angle showed that oil did not change the scaffold hydrophilicity. FTIR spectra confirmed that the oil finely coated the scaffold although there were some shifts in PLLA transition peaks when considering the vicinity of oil peaks with the PLLA peaks.

In this study, we have attempted to find a simple method of surface modification on an aligned PLLA/oil blend which can improve mechanical features, plasticity, and cell supports. This simple way of oil surface modification can elevate cytocompatibility and cell attachments of other types of scaffolds which are not biodegradable and have low cell attachment. Our results can specifically be used in special conditions of cell differentiation in the pancreatic region of a healthy rabbit. Differentiation of AMSCs to IPCs which need mechanical support and regulation of biodegradable scaffolds is subjected to the use of potent AMSCs as a dominant source of stem cells for tissue constitution. Numerous protocols have used various types of stem cells for induction to IPCs (20, 32). One study attempted to improve insulin secretion and optimization of produced IPCs (19). The present study used an efficient protocol to differentiate AMSCs to IPCs in contrast to other protocols. We have observed expression of *Pdx1* in the initial phase of IPCs induction. *Pdx1* is important in β cell development and function. *Pdx1* controls the regeneration of pancreatic β cells by regulating Insulin and other downstream genes. According to research, *Pdx1* expresses first followed by other IPC-related genes (19). A recent study demonstrated that in a specific injury model which induced β cell reconstitution (partial duct ligation), endogenous tissue-resident multipotent precursors were activated in a Ngn3 dependant manner (33). This study showed the expansion of Ngn3^+^ cells in the tissue throughout regeneration and the production of multiple islet cell types *in vitro* from Ngn3^+^ cells. Possibly, the originating IPCs might be Ngn3^+^ cells, as we, along with other researchers (34, 35), demonstrated the presence of Pdx1^+^/ Ngn3^+^ cells in the normal adult pancreas.

## Conclusion

Oil appeared to increase the adhesion area for attachment of differentiated cells onto the scaffold. Increase in cell attachment can be due to higher synthesis of intracellular molecular adhesions such as fibronectin and vitronectin. In the group with oil plus scaffold compared to scaffold alone, IPCs specific genes and protein expressions increased significantly after 21 days post-transplantation. These factors probably had a significant impact on the spatial structure, orientation, and quality of binding proteins such as fibronectin and vitronectin produced in the AMSCs after they were loaded on the scaffold. Use of scaffolding to differentiate MSCs in the insulin-secreting cells was assessed in previous studies. This study introduced a simple, efficient method to differentiate MSCs to IPCs without the use of an inducer in culture media.
